# A Novel Remedy for Hysteria

**Published:** 1849-07

**Authors:** 


*A Novel Remedy for Hysteria.*

At a late meeting of the Buffalo Medical Association, one of the members reported a case of hysteria, in which a method of treatment was pursued, which we do not recollect to have met with in our reading on this subject. The patient was a colored girl, about 18 years of age, and was subject to very frequent paroxysms of hysteria, for which, various remedies had been prescribed. On one occasion, the doctor, being satisfied that not an inconsiderable degree of the affection was due to willfulness, advised the mother to try the effect of a sound *spanking*. The mother, a robust washerwoman, having become annoyed, equally with the doctor, with the occurrence of the paroxysms on every slight occasion, was nothing loath to try this new expedient, and laid on the blows over the nates with right good will. The doctor remained to witness the faithful application of the remedy, and its results. In a short time the consciousness of the patient returned, the hysterical phenomena ceased, and she began to beg lustily that the treatment might be discontinued. She had no indications of the return of the malady, for a month—an unusually long interval—and then, upon the appearance of some of the premonitory symptoms, the bare intimation of a repetition of the same *methodus medendi*, proved sufficient to prevent an attack. The patient, in short, was effectually cured. We have frequently met with cases, in which our choice would have dictated some such remedial measure, but we have never ventured to follow the bent of our inclinations. The result, in this instance, certainly, should encourage the further trial of the plan, and we suggest it for the consideration of our professional brethren.

---

*Progress of Epidemic Cholera.*

This pestilential disease is on the increase at most of the different points of the country where it has made its appearance. At Cincinnati, Ohio, and St. Louis, Mo., it is prevailing to a great extent. As the sources of information with respect to its progress, are available, by means of the newspaper press, for all our readers equally with us, we shall not make any efforts to chronicle statistics, except in so far as the city of Buffalo, and its immediate vicinity, are concerned. The disease in this place is slowly and steadily on the increase. The Board of Health report, July

2d, during the preceeding 48 hours, 31 cases and 8 deaths.

The number of cases for the last two weeks, is 101; deaths during the same period, 38.

The whole number of cases since the appearance of the disease, May 30th, 1849, is 134; deaths, 51.

July 3d; number of cases during last 24 hours, 20; deaths, 5. *

